# Costs of mental health care resource use in people with obesity: A systematic review

**DOI:** 10.1371/journal.pone.0333123

**Published:** 2025-10-08

**Authors:** Nienke de Graef, Arianna Morris Gouveia, Aggie Paulus, Inge van der Putten, Emma Frew, Irina Pokhilenko

**Affiliations:** 1 Department of Health Services Research, Care and Public Health Research Institute (CAPHRI), Faculty of Health Medicine and Life Sciences, Maastricht University, Maastricht, The Netherlands; 2 Health Economics Unit, School of Health Sciences, College of Medicine and Health, University of Birmingham, Birmingham, The United Kingdom; 3 Faculty of Health Medicine and Life Sciences, School of Health Professions Education, Maastricht University, Maastricht, The Netherlands; 4 Centre for Economics of Obesity, Health Economics Unit, School of Health Science, College of Medicine and Health, University of Birmingham, Birmingham, The United Kingdom; Patuakhali Science and Technology University, BANGLADESH

## Abstract

**Introduction:**

Obesity is a complex condition with significant economic implications. Healthcare costs associated with obesity are spread across all categories of health services, including mental health care. However, little evidence is available regarding the magnitude and types of costs associated with mental health care resource use by people with obesity.

**Objectives:**

This systematic review aimed to synthesise the evidence on the costs of mental health care resource use by people with obesity.

**Methods:**

A systematic literature search was conducted in PubMed (Medline), Embase (Ovid), PsycINFO (EBSCO), and Econlit (EBSCO) based on PRISMA guidelines to identify cost-of-illness (COI) studies of obesity published in English since 2016. Peer-reviewed studies estimating obesity-related costs using primary or secondary data in children or adults were included. The COI studies quality tool by Schnitzler et al. was adopted to assess the methodological quality of studies. Data extracted included general study characteristics and costs associated with mental health care. Results were synthesised narratively.

**Results:**

A total of 5,565 records were identified post-duplication removal. Following selection, 33 COI studies were included, which mentioned mental health care costs; fifteen considered the monetary value of mental health care costs as separate costs in their analyses. The proportion of total annual healthcare costs attributable to mental health care ranged between 0.70% and 25.10%.

**Discussion:**

Our findings suggest that people with obesity incur substantial costs related to the use of mental health care, yet less than half of the included COI studies reported mental health care costs separately from total healthcare costs attributable to obesity. This highlights the importance of greater transparency and granularity when reporting costs. Furthermore, it is imperative to shed more light on the economic impact of co-morbid obesity and mental health. This research is essential for facilitating effective resource allocation and addressing the healthcare needs of this population.

## Introduction

Over the past few decades, the prevalence of obesity has been continuously rising [1–3]. Currently, almost a third of the world’s population is estimated to be overweight (defined as body mass index (BMI) of ≥ 25 kg/m^2^) or obese (defined as BMI ≥ 30 kg/m^2^) [4–7]. Obesity is a complex, multifactorial, and generally preventable condition that has put increasing pressure on the public health sector and societies worldwide [2,8]. Besides the increased risk of comorbidities (e.g., cardiovascular diseases and type 2 diabetes), obesity also has a significant societal and economic impact [7]. This impact includes higher healthcare consumption, reduced productivity, unemployment, and social disadvantages, such as stigma, limited access to opportunities, and social exclusion [9].

Obesity is associated with increased healthcare costs [10,11]. According to a study by Apovian [4], patients in the United States with obesity spend 42% more on medical expenses annually than those with a healthy weight. The direct healthcare costs associated with treating illnesses caused by obesity are the easiest to quantify. Okunogbe et al. [12] define direct medical costs as healthcare expenses incurred due to obesity-related diseases. These include treatments aimed at curing, rehabilitation, and preventing health issues, as well as ancillary services and medical supplies. Additionally, persons with obesity are more likely than people with a lower weight to use home healthcare services, have more outpatient appointments, be prescribed more drugs, be admitted more often to a hospital, and have more surgeries [12–14].

Obesity is also linked to the increased likelihood of developing mental health conditions [15,16]. Sarwer et al. [17] found that between 20% and 60% of people who are living with obesity experience mental health problems, which is higher compared to the general population. Baumeister et al. [15] confirm these findings, stating that individuals with obesity have higher rates of mood, anxiety, and somatoform disorders compared to those with a healthy weight. Conversely, mental health conditions, such as depression and anxiety, can also contribute to obesity through, e.g., emotional eating, reduced physical activity, and the side effects of certain medications [18–20]. Mental health and obesity are complex conditions, and their interplay depends on individual circumstances, including epigenetic and environmental factors [21]. Moreover, numerous factors influence the bidirectional relationship between obesity and mental health [11], including body dissatisfaction, low self-esteem [22–24], and the persistent stigma associated with obesity, which has detrimental effects on individuals’ physical, psychological, and socioeconomic health [25]. Furthermore, multiple mechanisms have been found to contribute to the link between poor mental health and obesity [26], such as the tendency to seek instant gratification via food due to the social stigmatisation of obesity [27,28] and hormonal changes caused by chronic stress and depression [29].

Evidence supports the view that the indirect costs of obesity account for a more significant proportion of the total costs of overweight and obesity [12]. Previous studies have demonstrated that as BMI rises, sick days, temporary leave, and other indirect costs rise, too [14,30,31]. Nevertheless, direct medical costs continue to burden healthcare systems, as indirect and direct costs both rise with increasing BMI [32,33]. Simon et al. [34] found that the increased costs associated with obesity are distributed across all major health service categories, including mental health care. In 2017, the systematic review of cost of obesity studies by Tremmel et al. [35] reported that only four out of 23 included publications considered mental health care costs, highlighting the importance of considering these costs in future studies [35].

Although the economic burden of obesity is well researched, evidence on the mental health care costs associated with obesity remains sparse. This gap has implications for mental health service planning, providing a strong justification for this systematic literature review. Understanding the mental health care costs incurred by people with obesity helps assess the economic burden and optimise resource allocation. Therefore, this review aimed to synthesise and assess the methodological quality of the economic evidence on mental health care resource use of people with obesity by reviewing cost of obesity studies. With the growing pressure on healthcare systems worldwide and the increasing rates of obesity and mental health conditions [1–3,36], this study evaluates the strength of current evidence to inform policy and practice and provides direction for future research.

## Methods

This systematic literature review aimed to compile the mental health-related costs in people with obesity from relevant studies to help facilitate policy-makers and healthcare practitioners’ access to available evidence [37]. The study was designed according to the methodological guidance for conducting systematic literature reviews of health economics studies, as outlined by Mastrigt et al. [38] and Thielen et al. [39]. The extension by Preferred Reporting Items for Systematic Reviews and Meta-analysis (PRISMA) was used as a basis for reporting the results [40]. The completed PRISMA checklist is available in the Table in S1 Table. Additionally, the PICOS framework was used to shape the research question, the search strategy, and the inclusion and exclusion criteria.

### Eligibility criteria

We included cost-of-illness (COI) studies that estimated obesity-related costs using primary data (i.e., newly collected data) or secondary data (i.e., already existing data) in both children and adults, regardless of the analysis perspective and type of comparator. There were no restrictions on the country of origin of the studies. Eligibility was limited to peer-reviewed journal articles published in the English language in or after 2016, as a similar review by Tremmel et al. reviewing cost of obesity studies included publications up to 2016 [35].

We excluded full economic evaluation articles and publications that did not mention direct or indirect obesity-related costs or focused only on obesity-related comorbidities, making comparison difficult because costs could be over- or underestimated compared to considering the total costs of obesity. Additionally, we excluded systematic reviews, non-peer-reviewed sources (e.g., grey literature), letters to the editor, conference abstracts, and poster presentations.

All studies meeting the inclusion criteria were included in the synthesis regardless of whether they included mental health care costs. The identification and assessment of studies that specifically considered and quantified mental health care costs were conducted as part of a later multi-stage screening and analysis process.

### Information sources and search strategy

Four databases were searched at the beginning of May 2023, and the search was updated at the end of December 2024. The databases included PubMed (Medline), Embase (Ovid), PsycINFO (EBSCO), and Econlit (EBSCO). These databases were selected as they provided adequate and efficient coverage of the topic for this review [39,41,42]. PubMed and Embase are widely used as comprehensive databases; to complement the search, PsycINFO and Econlit were added due to their focus on mental health and economics literature, respectively.

The initial step was formulating a list of search terms to identify relevant studies. A comprehensive compilation of synonyms for the identified keywords was assembled. In addition, Medical Subject Headings (MeSH) terms were incorporated into the search list of PubMed. Examining keyword lists from publications identified in an initial PubMed search, along with discussions with an information specialist from Maastricht University’s library, helped identify additional search terms. This process ensured the inclusion of all relevant terms necessary for a comprehensive search strategy [35,39,43]. The data in S1 Text contains the search term list for each database and search strategy, along with the number of records identified.

### Identification and screening of relevant studies

Results from the database searches were uploaded to EndNote 21.5 for duplicate removal. Further duplicates were removed manually or on Rayyan [44], as reference software is not always reliable [38]. Included articles were uploaded onto Rayyan, an online systematic review management platform [44]. All articles were blind double-screened by two reviewers (NdG and AMG). Eligibility criteria were first applied to the titles and abstracts of the studies. Then, the full texts of the remaining studies were thoroughly read and screened using the same approach. Any discrepancies were discussed until consensus was reached after every 25% for the title/abstract screening stage and after 50% and 100% for the full-text screening stage. A third stage of screening was conducted, where the COI studies were reviewed again to determine whether they specifically considered mental health care costs.

A recently published systematic review by Nagi et al. was identified, which considered the cost of obesity across a broad range of cost categories, but did not focus specifically on mental health-related costs [45]. The included studies of that review were compared with those identified through our search strategy to further ensure no relevant articles had been missed. This comparison did not yield any additional studies. Conversely, the studies identified in the systematic review by Tremmel et al. [35] were not compared to our included studies, as we specifically chose to focus on evidence published after 2016 to better reflect current epidemiological trends and cost data.

### Data extraction and analysis

The data extraction form was created with input from all co-authors. It contained sections on general and specific study characteristics relevant to the research question [46], and was completed by two reviewers (NdG and AMG). The general characteristics included country, time frame of the study, type of data (primary or secondary), data sources, and how data were synthesised. This data was summarised based on how the authors within the included articles reported it. The data on the economic characteristics included the chosen perspective, the types of costs, the inclusion of mental health care costs, and the mental health services included in these costs. Additionally, a distinction was made between the articles that mentioned the monetary value of mental health care costs and those that did not. If mental health care costs were reported separately, they were extracted and recalculated to Euros (2024) [47]. When the information was available, we also calculated the proportion of mental health-related costs relative to total healthcare costs as follows: the total mental health care costs reported divided by the total healthcare costs reported, multiplied by 100. A second reviewer (IvdP) verified these calculations to ensure accuracy.

### Narrative synthesis approach

As meta-analysis was not appropriate due to substantial heterogeneity in study designs, populations, and outcome measures, we conducted a synthesis without meta-analysis (SWiM), following the SWiM reporting guideline [48]. We grouped studies based on whether they reported the monetary value of mental health-related costs separately, as a proportion or increase of total obesity-related costs, or as part of overall costs. Data were presented in summary tables to facilitate transparency and comparison across studies. Where possible, we converted reported costs to a common currency and year to enable descriptive comparison. Findings were synthesised narratively using textual summaries. We highlighted patterns in cost reporting and identified inconsistencies in how mental health-related costs were defined or calculated. A quality appraisal of included studies was conducted using a consensus-based checklist for COI studies by Schnitzler et al. [49] to inform the interpretation of findings, though it did not guide study inclusion or weighting. We did not apply any weighting of studies by quality or size, and all were included equally in the synthesis. Although we noted variation in study design and cost reporting, no formal investigation of heterogeneity was conducted.

### Quality appraisal

A consensus-based checklist for the critical appraisal of COI studies by Schnitzler et al. [49] was adopted to assess the methodological quality of the included studies and to help guide the research recommendations. The article of Schnitzler et al. [49] contains this COI consensus-based checklist, which includes relevant questions related to study characteristics, methodology, and cost analysis, as well as results and reporting of COI studies. One reviewer (NdG) independently conducted the quality appraisal of all included studies using the appraisal tool, while a second reviewer (AMG) assessed a randomly selected 25% of the articles.

## Results

**Fig 1:**
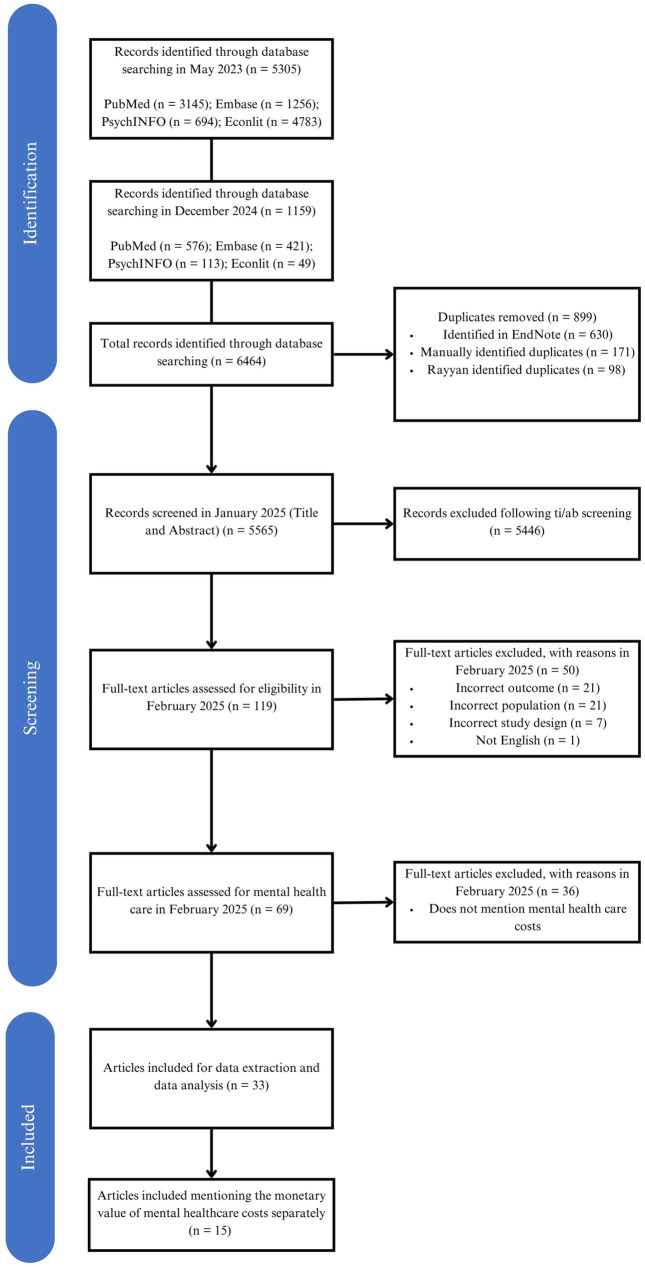
PRISMA flow diagram of the study selection process.

The database search conducted in May 2023 resulted in 5,305 hits, and the updated search in December 2024 resulted in 1,159 hits. After deduplication, 5,565 articles remained. The titles and abstracts of these articles were screened against the inclusion and exclusion criteria, and 5,446 articles were excluded. The full texts of the articles were evaluated based on the eligibility criteria, which resulted in the exclusion of 50 articles. A third-stage screening was then conducted on the remaining 69 studies to determine whether they included mental health care costs. As a result, 33 articles were included in the review for data extraction and analysis [5,50–81]. Fig 1 shows the PRISMA flowchart, illustrating the selection process.

### General study characteristics

The Table in S2 Table contains the data extraction results describing the general characteristics across all included COI studies. Table 1 summarises this information. Of the studies reviewed, 24 estimated the costs of obesity in adults [5,50–53,56–60,62–65,69–71,73–75,77,78,80,81], four focused exclusively on children and/or adolescents [55,67,68,79], and five included adults, children, and/or adolescents [54,61,66,72,76].

**Table 1:** General study characteristics.

Study characteristics	Category	n
Population	Adults	24
	Children and/or adolescents	4
	Both	5
Continent	Europe	20
	Asia	2
	North America	7
	South America	1
	Africa	2
	Oceania	1
Methods for calculating costs	Top-down approach	15
	Bottom-up approach	17
	Varied	1
Data collection approach	Retrospective cost approach	29
	Prospective cost approach	3
	Varied	1
Measure of disease	Prevalence-based	29
	Incidence-based	2
	Both	1
	Varied	1
Type of data	Secondary data	29
	Secondary and primary data	3
	Primary data	1
Definition of obesity	WHO classification	16
	Other classification	16
	No definition	1
Comparison group	Nonobese or healthy-weight persons	17
	N.M. or none	14
	N.R.	2

N.M. = Not mentioned and N.R. = Not relevant.

There were twenty studies from Europe [5,51,53,54,56,58–60,62,63,65,66,72,73,75,76,78–81]. Seven studies were conducted in North America [52,57,64,67,68,70,77]. Two studies were conducted in Asia [71,74], two were conducted in Africa [50,69], one was conducted in South America [61], and one included data from Oceania [55].

Fifteen studies [50,51,56–59,61,62,64–68,72,78] calculated the costs attributable to obesity using a top-down (population-based) approach. The top-down approach estimates disease-specific costs using total health expenditures and disease-specific rates [82]. Seventeen studies [5,52–55,60,63,69–71,73–75,77,79–81] adopted a bottom-up approach. The bottom-up approach estimates costs by considering the number of healthcare inputs and calculating expenses based on the individual costs of the delivered service [82]. A varied approach was utilised in one study by Steinl et al. [76].

Out of the 33 included studies, 29 articles [50–58,60–64,66–75,77–81] were retrospective analyses; 26 [50–53,55–58,61–64,66–71,73–75,77–81] of those were prevalence-based, two were incidence-based [54,72], and one was both prevalence- and incidence-based [60]. Three studies [5,59,65] were prospective and prevalence-based. Finally, one study had a varied epidemiological and data collection approach [76].

Almost all articles used secondary data to estimate costs; only the study by Williamson et al. used primary data only [80]. Besides using secondary data, the studies of Lartey et al. and Hecker et al. also collected primary data using questionnaires, and the study of Aboulghate et al. collected primary data via interviews [5,50,69].

Sixteen articles [5,50,51,58–62,64–66,69,71,75,78,81] used the World Health Organization (WHO) classification to define obesity [7]. Ten other articles [52–54,57,63,70,73,74,77,80] used a different classification than the WHO one, but often closely related to it. Six articles [55,67,68,72,76,79] did specify that their study was about obesity, but the population was predominantly children, so they used a different classification than the WHO. One article [56] did not define obesity clearly.

Fourteen studies [5,50,56,61,63,66,68,70–73,76,77,80] did not mention a comparison group; however, they occasionally compared their results of people with obesity to those of people with overweight. Seventeen studies [51,54,55,57–60,62,64,65,67,69,74,75,78,79,81] did use a comparison, such as nonobese or healthy-weight persons. Two studies [52,53] used an irrelevant comparator, like in the study of Ard et al., which compared individuals who underwent bariatric surgery against those who did not undergo surgery, or in the study of Atella et al., which compared normal glucose tolerance, impaired fasting glucose and diabetes mellitus.

### Economic characteristics of included studies

The study perspective determines which healthcare costs and benefits should be included in economic analyses [83]. The most used perspectives were third-party payer [56,59,63,67–71,73,74,77,79] used in twelve studies and healthcare system [50,52–55,57,58,61,64,65,76,78,80] used in thirteen studies, which only included the direct healthcare costs associated with obesity. These costs varied between studies but included costs for inpatient care, hospital-based outpatient care, primary care, pharmaceutical costs, municipal care, palliative care, monitoring, adverse events, nursing, diagnostic tests, treatment costs, and costs related to the purchase of supporting devices. Seven studies utilised a societal perspective [5,51,62,66,72,75,81] and in addition to direct costs, also accounted for productivity losses. A life-cycle [60] perspective was used in one study. A life-cycle perspective, more commonly used in health-technology assessments [84] is similar to a societal one, except it considers costs over a patient’s life, such as long-term health, cumulative productivity losses and mortality [85]. Table 2 contains a detailed description of the economic characteristics of the included articles.

**Table 2:** Mental health care costs.

Author, Publication, Year, Country	Perspective	Cost categories	Mental health care service or condition costs considered	Total monetary value of mental health care costs associated with obesity per year	Monetary value of total mental health care costs converted to 2024 Euros**	% of mental health care costs in relation to the total healthcare costs
**Studies which included mental health-related costs separately in monetary values**						
M. Aboulghate et al., 2021, Egypt [50]	Healthcare system	Direct medical costs	Annual societal costs of depression treatment attributable to obesity	440,000,000 Egyptian Pounds	118,200,023.12	0.70
E. Andersson et al., 2022, Sweden [51]	Societal	Direct medical costs and lost production due to absence from work	The healthcare costs for society for obesity and depression	7,403,000 Euros	9,510,963.14	2.11
M. Borges et al., 2024, Portugal [56]	Portuguese national health service	Direct medical costs (inpatient and specialised outpatient care, primary care, and pharmacological treatment)	Depression is included as an ORC with costs for society separated by service	26,843,000 Euros	41,188,169.13	2.34
S. Butalia et al., 2023, Canada [57]	Healthcare system	Direct medical costs	Healthcare costs of depression for society	256,120,509 Canadian Dollars	202,183,932.06	12.30
Y. Gil-Rojas et al., 2019, Colombia [61]	Healthcare system	Direct medical costs	Costs of depression per patient per year and total annual impact	113,057,645 US Dollars	113,649,257.02	5.24
J. Hecker et al., 2022, The Netherlands [5]	Societal	Direct medical, direct non-medical, and indirect costs	Mental health institution, psychologist, psychotherapist, psychiatrist – practice, psychologist, psychotherapist, psychiatrist – hospital services were considered as part of direct medical costs per person	315.2 * Euros	371.74	10.84
K.E. Kyler et al., 2023, United States [68]	Third-party payer	Direct medical costs	Hospitalisations, drugs, and outpatient costs associated with mental health conditions.	90,557.02 US Dollars *	89,333.60	5.61
J. Pearson-Stuttard et al., 2024, United Kingdom [63]	UK NHS	Direct medical costs	Depression average annual costs were reported per weight class	Overweight: 1,616 Pounds Sterling Obesity I: 1,801 Pounds Sterling Obesity II: 2,105 Pounds Sterling Obesity III: 2,306 Pounds Sterling	Overweight: 2,244.19 Obesity I: 2,501.10 Obesity II: 2,923.28 Obesity III: 3,202.41	N.A.
S. Reitzinger et al., 2024, Austria [72]	Societal	Direct medical and indirect (absenteeism) costs	Societal costs of “mental and behavioural disorders,” reported separately by three obesity classes	Obesity I: 89,184,600 Euros * Obesity II: 41,275,200 Euros * Obesity III: 14,263,200 Euros *	Obesity I: 103,571,904.30 Obesity II: 47,933,735.92 Obesity III: 16,564,146.56	6.00
M. Spanggaard et al., 2022, Denmark [75]	Societal	Direct medical and indirect costs	Direct psychiatric expenditure per person	576 Euros *	679.33	11.45
D. Steinl et al., 2024, Switzerland [76]	Healthcare system	Direct medical costs	Annual total depression costs attributed to overweight/obesity	595.000.000 Swiss Francs	424,767,269.46	11.71 - 16.86
A. Vesikansa et al. 2023, Finland [78]	Healthcare system	Direct medical costs	Visits for psychiatric disorders per person	215 Euros	228.21	8.06
A.H. Wijga et al., 2018, The Netherlands [79]	Third-party payer (Dutch healthcare insurers)	Direct medical costs	Mean mental health care utilisation and costs per person reported	1528 Euros	22,516.34 *	25.10
**Studies which included mental health-related costs separately as a proportion or increase of total obesity-related costs**						
V. Gorasso et al., 2022, Belgium [62]	Societal	Direct medical costs and indirect costs	‘Serious gloom or depression’ included as an ORC, but the costs were reported as ratios	N.A.	N.A.	N.A.
L. Kompaniyets et al., 2020, United States [67]	Hospital payer	Direct medical costs	Costs associated with mood disorders and schizophrenia. Costs reported as an increase on baseline (obesity only)	+1,059.7 US Dollars	+1065.25	N.A.
C. Rudisill et al., 2016, England [73]	UK NHS	Direct medical costs	Costs associated with depression. Costs reported as an increase on baseline (obesity only)	+1044 Pounds Sterling	+1593.23	N.A.
H.J. Song et al., 2018, South Korea [74]	Third-party payer	Direct medical costs	Depression was included as an ORC, but costs were only reported as a ratio	N.M.	N.A.	N.A.
**Studies which considered mental health care costs as part of total obesity costs but did not report them separately**						
J. Ard et al., 2023, United States	Healthcare system	Direct medical costs	Depression was included as an ORC, but costs for patients with depression and obesity were not reported separately	N.M.	N.A.	N.A.
V. Atella et al., 2023, Italy [53]	Healthcare system	Outpatient costs (diagnostic tests, specialist visits, and drugs)	Depression was included as an ORC, but costs for patients with depression and obesity were not reported separately	N.M.	N.A.	N.A.
V. Atella et al., 2024, Italy [54]	Healthcare system	Outpatient costs (diagnostic tests, specialist visits, and drugs)	Depression was included as an ORC, but costs for patients with depression and obesity were not reported separately	N.M.	N.A.	N.A.
N. Black et al., 2018, Australia [55]	Healthcare system	Direct medical costs (primary care, outpatient hospital services and prescription drugs)	Mental health-related costs were considered as part of the total costs, but not reported separately	N.M.	N.A.	N.A.
A. Colao et al., 2017, Italy [58]	Healthcare system	Direct medical costs	Costs related to depression and antidepressant use	N.M.	N.A.	N.A.
K. Destri et al., 2024, Portugal [59]	Portuguese national health service	Hospitalisation costs	Mental disease was included as part of the total obesity costs	N.M.	N.A.	N.A.
T. Effertz et al., 2016, Germany [60]	Life-cycle	Direct medical, indirect, and societal costs	Panic disorder ‘[episodic paroxysmal anxiety]’ included as part of total obesity costs	N.M.	N.A.	N.A.
P. Kamble et al., 2018, United States [64]	Healthcare system	Direct medical costs	Antidepressants and antipsychotic medication utilisation were included, but the costs were not reported separately	N.M.	NA.	N.A.
S. Kent et al., 2017, England [65]	Healthcare system	Direct medical costs	Self-reported depression and anxiety costs were included but not reported separately	N.M.	N.A.	N.A.
J. Kjellberg et al., 2017, Denmark [66]	Societal	Direct medical and indirect costs	Psychiatric inpatient and outpatient costs were included in total healthcare costs.	N.M.	N.A.	N.A.
S.T. Lartey et al., 2020, Ghana [69]	Government and patient	Direct medical costs	Depression counted as one of the chronic disease comorbidities, but costs were not reported separately	N.M.	N.A.	N.A.
S. Musich et al., 2016, United States [70]	Government and patient	Direct medical costs	Self-reported mental health costs were included, but costs were not reported separately	N.M.	N.A.	N.A.
X, Qin et al., 2016, China [71]	Government and patient	Direct medical costs	Depression was included as an ORC, but costs for patients with depression and obesity were not reported separately	N.M.	N.A.	N.A.
B.T. Suehs et al., 2017, United States [77]	Third-party payer (Medicare)	Direct medical costs	Antidepressant utilisation was reported, but costs for patients with depression and obesity were not reported separately	N.M.	N.A.	N.A.
K. Williamson et al., 2023, Scotland [80]	Healthcare system	Direct medical costs	Mental health care use was considered, but costs were not reported separately	N.M.	N.A.	N.A.
N. Yates et al., 2016, Germany [81]	Societal	Direct medical and indirect costs	Psychotherapists/psychiatrists’ annual costs for medical service utilisation	N.M.	N.A.	N.A.

*   = Calculated by aggregating costs or adjusting to an annual basis. ** = Converted with a web-based tool by Shemilt et al. (2010) [47] on March 14^th^ 2025. ORC = obesity-related condition, N.M. = Not mentioned, N.A. = Not applicable.

### Mental health care services

There was a substantial variation in the types of mental health care services reported and how they were described in the included studies, as seen in Table 2. Several articles [5,66,75,78] mention including the costs associated with the use of specific services such as psychiatric inpatient and outpatient services, or visits to a psychologist or psychiatrist. Other articles provided less detail about the type of services incorporated in the mental health care costs, with seven papers [50,51,56,57,62,74,76] delineating aggregated costs for society for obesity and depression, while three other studies mentioned the costs of depression as an obesity-related comorbidity [61,63,73]. Kompaniyets et al. [67] examined the incremental costs associated with a secondary diagnosis of obesity and a primary diagnosis of mood disorders, or schizophrenia, without specifying what these costs entailed. Kyler et al. (2023) discussed several mental health conditions combined and mentioned hospitalisations and pharmacy costs, and Reitzinger et al. included mental and behavioural disorders separated into three obesity classes [68,72].

### Mental health care costs

Fifteen studies [5,50,51,56,57,61,63,67,68,72,73,75,76,78,79] included estimates of the monetary value of mental health care costs, with most focusing on the costs associated with depression. Several studies examined the total annual costs of depression attributable to obesity at a societal level. Aboulghate et al. [50] reported the annual cost of depression related to obesity around 114 million Euros, while Andersson et al. [51] found an annual cost of around 9.5 million euros. Additionally, Borges et al. [56] estimated a total cost of around 41.2 million attributable to depression and Butalia et al. [57] calculated the population level total healthcare costs of obesity among those living with severe obesity to be around 202.2 million Euros. Similarly, Steinl et al. [76] reported 424.8 million Euros in annual costs.

Beyond these overall costs, three studies also provided estimates for annual per-person costs of depression. Pearson-Stuttard et al. [63] broke down the average annual costs by weight class, reporting costs of 2,244.19 Euros for overweight individuals, 2,501.10 Euros for Obesity I, 2,923.28 Euros for Obesity II, and 3,202.41 Euros for Obesity III. Rudisill et al. [73] estimated a mean increase in annual patient costs if depression was present of 1593.23 Euros, while Gil-Rojas et al. [61] reported costs of 678.71 Euros for adults, 98.13 Euros for children, and an annual total impact of more than 113.6 million Euros.

The costs associated with mental health care were also explored in children and adolescents with obesity in several other studies. Kompaniyets et al. [67] estimated mental health care related costs among young people aged 2–19 years with both a primary diagnosis of mental health and/or substance abuse (MHSA): mood disorders and MHSA: schizophrenia. They found that mood disorders, with a positive cost increment of 666.17 Euros per year, had a significant association with having more costs when obese, while for schizophrenia, with a positive cost increment of 399.08 Euros per year, there was no significant association found. In a study by Kyler et al. [68], inpatient healthcare hospitalisation, pharmacy costs, and outpatient costs regarding mental health conditions were estimated at 89,333.60 Euros per year for children aged 2–17 years. Wijga et al. [79] reported a mean total annual expenditure of 22,516.34 Euros for mental health care use by children aged 11 and 14 years. Reitzinger et al. [72] looked at the broader costs of mental and behavioural disorders in both adults and children, estimating the costs by obesity class at 103.57 million Euros for Obesity I, 47.93 million Euros for Obesity II, and 16.56 million Euros for Obesity III.

Additionally, some studies provided estimates associated with the use of specific mental health services used by people with obesity. Spanggaard et al. [75] estimated the average annual direct healthcare costs per person. These costs included 455.24 Euros for psychiatric inpatient hospitalisation, 171.01 Euros for psychiatric outpatient visits, and 53.07 Euros for psychiatric medication. Hecker et al. [5] estimated the average healthcare costs over six months for people with overweight or obesity, including 109.55 Euros for mental health institutions, 51.92 Euros for psychologists, psychotherapists, and psychiatrist practices, and 24.40 Euros for psychologists, psychotherapists, and psychiatrist hospitals. Lastly, Vesikansa et al. [78] discussed the annual costs of psychiatric disorders in people with obesity, amounting to 228.21 Euros.

### Proportion of mental health care costs

The study by Aboulghate et al., Andersson et al., and Reitzinger et al. were the only articles that discussed how much of the total healthcare costs were attributed to mental health care costs [50,51,72]. This percentage was not possible to estimate for the studies by Kompaniyets et al. and Rudisill et al. [67,73] because the authors described the mental health care costs as incremental increases due to obesity rather than providing absolute cost values. This approach makes it unclear how much of the total mental health care costs are attributable specifically to obesity. The proportion of mental health care attributable to obesity could also not be estimated in Pearson-Stuttard et al. [63]. For the other nine articles [5,56,57,61,75,76,78–80], estimations were made based on the total healthcare costs of obesity and the mental health care costs described in the papers. The percentages ranged between 0.70% and 25.10% over the twelve articles [5,50,51,56,57,61,68,72,75,76,78,79].

### Quality appraisal

The table in S3 contains the results from the COI critical appraisal evaluation tool by Schnitzler et al. [49]. On average, the evaluation tool found the quality of the COI studies to be more favourable than unfavourable across all 33 articles. Notably, all articles had a well-defined population and objective except for the study by Atella et al. (2024) [54], which lacked a well-defined research question.

One recurring issue was that the studies often did not explicitly state the chosen study perspective. This was evident in studies by Ard et al. and Kyler et al. [52,68]. However, in most studies, the perspective could still be inferred from how the costs were calculated, as seen in Aboulghate et al. and Atella et al. [50,53]. Similarly, abstraction was often required when assessing the epidemiological, costing, and data collection approaches. While most studies did report the epidemiological approach [5,56,59–65,69–81], they did not always specify whether their costing approach was top-down or bottom-up, nor did they consistently indicate whether the study was conducted prospectively or retrospectively.

All included studies identified and measured all the components of resource use. Additionally, the time horizon was almost always specified, though not always justified by the authors. Future costs were only discounted in the study by Ard et al. and Effertz et al. [52,60]. Furthermore, sensitivity analyses were conducted in only nine studies [55,59,65,69,71,72,75,80,81], and most of these studies justified the variables subjected to this sensitivity analysis, except for the study by Destri et al. [59].

Additionally, all studies included analyses of relevant subgroups, represented the results transparently by cost category and discussed the generalisability of study results. However, the study by Wijga et al. [79] did not compare their study results with those of other groups or settings.

Moreover, all authors acknowledged some limitations within their studies. Most studies also discussed distributional issues, except for Aboulghate et al. and Wijga et al. [50,79].

Finally, ethical considerations were generally stated as not applying to the study or omitted entirely from the paper. Additionally, twelve studies disclosed a potential conflict of interest [50–52,56,57,63–66,75–77], whereas four articles did not report any information regarding conflicts of interest [55,68,71,78].

## Discussion

This review synthesised available evidence for the mental health care costs of people living with obesity. A total of 33 cost-of-illness studies published since 2016 were identified which consider mental health care costs in relation to obesity. Of these, fifteen explicitly reported the monetary costs of mental health care use by people with obesity. Mental health care use was estimated to contribute between 0.70% and 25.10% to the total healthcare costs associated with obesity.

### Reflection on main results

There was variation among the 33 reviewed studies regarding the magnitude of mental health care costs, ranging between 0.70% and 25.10%. Different countries and populations were considered as well as a wide range of methodologies were adopted, resulting in varying financial impacts on society. We found inconsistencies in the description and type of mental health care services, the costs of which were estimated in the included studies. Most service descriptions do not adequately describe the nature of service provision, which challenges the comparability across studies and makes it more difficult for policymakers and healthcare professionals to understand the distribution of costs across specific services, highlighting the need for more transparent reporting in future COI studies in this research area. This variation in the proportions of mental health care costs may also be explained by the differences in healthcare systems and spending on mental health care services in different countries. For example, in Egypt, around 1% of healthcare spending is allocated to mental health care services, with most services funded from out-of-pocket payments, not captured in direct medical costing studies [86]. In comparison, the Netherlands spends 27% of its healthcare spending on mental health care services [87].

Depression was the most commonly reported condition in the reviewed studies to be associated with obesity, and the costs of depression were most frequently reported compared to other conditions. When comparing the results of this study to the systematic review and meta-analysis by Pereira et al. [88], it is apparent that there is increasing evidence that obesity is linked with depression. Depression has been associated with overweight and obesity in people as young as 10 years and older [61]. Additionally, Ling et al. [89] identified that being overweight during childhood and adolescence is a strong indicator of adult obesity, bringing about substantial immediate and lasting physical and psychological risks, including a reduction in mental well-being. This evidence underscores how obesity and mental health are critical public health issues which should be tackled in both adults and young people. With the increase in childhood obesity and the rate of mental health conditions, more robust economic evidence is required to support resource allocation in the future.

A person with obesity belonging to a patient organisation ‘European Coalition for People living with Obesity’, was consulted in the process of this study. They argued that healthcare providers should consider the bidirectional relationship between obesity and mental health, as well as the associated stigma in their care, which would also increase patients’ engagement. By adopting a comprehensive approach that considers these factors, healthcare providers can demonstrate greater sensitivity towards individuals with obesity, acknowledge the associated stigmas surrounding weight and mental health issues, and take a tactful approach to address these concerns at every level of care.

### Reflection on quality appraisal

The consensus-based checklist for COI studies by Schnitzler et al. [49] did not suggest a proportion of criteria required for a study to be deemed good or poor quality. Therefore, the results of the quality appraisal were analysed narratively to provide an impression of the comprehensiveness and methodological rigour of the included studies [49].

A key observation from this quality appraisal process is that specific criteria from the evaluation tool were poorly scored across most studies. For example, many studies poorly reported their costing and data collection approaches. However, overall, the methodological quality of the included studies was favourable. The variability in quality highlights the need for greater consensus in COI study reporting, ensuring that results can be trusted by decision-makers.

Moreover, the quality appraisal tool does not account for how well subcategories of costs are captured, in the case of our study, mental health care costs within the overall healthcare costs or direct medical costs of obesity. To overcome this, the included studies were grouped by how they reported mental health care costs, which allowed for a better understanding of how frequently mental health care costs are presented disaggregated in existing literature.

### Comparisons with existing literature

Until recently, there was no updated systematic review of the economic burden of obesity since Tremmel et al. [35] in 2017. In 2023, an update was provided by Nagi et al. [45]. However, our review differs from both as it focuses on the mental health care costs in people with obesity. In the review by Tremmel et al. (2017), only four studies were identified which included costs associated with mental health care resource use [35]. This is in line with the growing awareness of the economic burden of mental health conditions and the link between mental health and physical health conditions, including obesity. The review by Nagi et al. [45] did not report how many of their included studies considered mental health care costs.

Both previous reviews noted the variety of evidence, with Tremmel et al. highlighting that greater consensus was needed on estimating the economic burden of obesity to ensure greater comparability of the evidence [35]. Our findings support this conclusion.

### Strengths and limitations

There are several strengths of this review. Firstly, to our knowledge, this is the first systematic review to examine the mental health-related costs associated with obesity. Secondly, PRISMA guidelines were followed to ensure clarity and reproducibility. Thirdly, the selection of databases and search strategies was comprehensive to increase sensitivity, ensuring that fewer relevant studies were missed. Additionally, no restrictions were placed on the setting of studies to maximise the variety of evidence collected, improving on the review by Tremmel et al. [35]. Moreover, screening and data extraction were conducted by two independent reviewers, which limited selection bias. Finally, where monetary costs of mental health service use were reported separately, those were recalculated to 2024 Euros to facilitate comparability.

On the other hand, there are also some limitations worth considering. Although a strength of this review is the breadth of evidence collected, this also meant that meta-analysis was not possible due to the variety of settings and methodological approaches. Therefore, this review has provided a narrative synthesis of available evidence. Although we conducted a formal quality appraisal of individual studies using a consensus-based checklist for COI studies by Schnitzler et al. [49], we did not apply the GRADE framework to assess the overall certainty of the evidence. Furthermore, this systematic review only focused on one side of the bidirectional relationship between obesity and mental health and did not consider the obesity-related costs in COI studies of mental disorders, which could provide a more comprehensive picture.

Additionally, the estimated percentage of mental health care costs as a proportion of total healthcare costs did not account for the variation (standard deviation) in mental health care resource use reported within the included studies. So, in reality, the range is likely to be greater than between 0.70% and 25.10%. Finally, although a pre-registered protocol was not in place, a structured and transparent approach was followed throughout to reduce the risk of bias.

### Recommendations for policy

Less than half of the eligible studies in this review have provided precise estimations of mental health care costs incurred by people with obesity. Consequently, it is imperative to enhance awareness of mental health care costs attributed to obesity, thereby enabling healthcare practitioners and policymakers to allocate resources more efficiently. In this regard, it is crucial to recognise the diverse range of mental health issues that can contribute to obesity and how obesity can contribute to the development and progression of mental health conditions. Emphasising mental health care and implementing preventative measures for mental health conditions would be advantageous in mitigating the prevalence of obesity and its associated costs. Furthermore, healthcare practitioners across all levels of care should emphasise addressing the stigmatisation surrounding both obesity and mental health issues, fostering improved patient engagement and more effective care provision for this specific population group. Ensuring the delivery of appropriate levels of care makes obtaining more accurate estimations of mental health care costs more feasible, thereby rectifying the prevalent issue of underestimation of mental health care costs in people with obesity.

### Recommendations for future research

Further research is needed to gain high-quality evidence on the mental health care costs for people with obesity. Future research should clearly define and transparently report what mental health care service costs are included to allow for better comparison between studies. Additionally, researchers should try to ascertain whether increased mental healthcare costs are a result of obesity or obesity is a result of a pre-existing mental health condition. Research into this bidirectional relationship will provide policymakers with a better understanding of where mental health care resources will likely be most cost-effective. However, it is essential to acknowledge that this line of research encounters inherent difficulties due to the prevailing stigma surrounding both obesity and mental health issues and its bidirectional relationship, thereby making it a complex and multifaceted topic to explore. Nevertheless, this topic holds significant implications for public health, and a comprehensive approach acknowledging the experiences of people who have both mental health conditions and obesity can potentially allow for valuable insights.

## Conclusion

People with obesity are more likely to experience mental health conditions leading to higher consumption of healthcare resources. Yet, the research on the costs associated with mental health care use by people with obesity is scarce. This review synthesised the evidence on mental health care costs incurred by people with obesity by reviewing recent cost-of-obesity studies. The study highlights variability in the types of costs considered, the methods adopted, and the overall magnitude of costs. Due to the diversity of included studies, mental health care is estimated to be responsible for between 0.70% and 25.10% of obesity-related costs. The results also underscore the importance of further research in this area, especially in the child and adolescent population, considering the recent rise in childhood obesity and mental health conditions in young people. Furthermore, the observed methodological variability points to the need for a more comprehensive, standardised approach to studying the relationship between obesity and mental health and associated costs to inform future public health policies.

## Supporting information

S1 Table Completed PRISMA checklist. (DOCX)

S1 Text Search term lists and strings. (DOCX)

S2 Table Data extraction sheet general characteristics. (DOCX)

S3 Table Quality appraisal results. (XLSX)
