# Supplementary material for: Costs of mental health care resource use in people with obesity: A systematic review
Source: PLoS One. 2025 Oct 8;20(10):e0333123. doi: 10.1371/journal.pone.0333123 (PMC12507204; doi:10.1371/journal.pone.0333123)
Supplement: S1 Text — (DOCX) [file pone.0333123.s002.docx]

S2 Text. Search term lists and strings

**Search term list Pubmed** (32, 36, 40) Keyword table PubMed 02/05/2023 and 19/12/2024

| **Keyword 1** | **Keyword 2** |
| --- | --- |
| Obesity (MeSH Terms) | Cost of illness (MeSH Terms) |
| Obes* (tiab) | Economics (MeSH Terms) |
| Overweight (MeSH Terms) | Economic outcomes (tiab) |
| BMI (tiab) | Costs and cost analysis (MeSH Terms) |
| Body mass index (MeSH Terms) | Health care costs (MeSH Terms) |
| Obesity, abdominal (MeSH Terms) | Health expenditures (MeSH Terms) |
| Abdominal obesity metabolic syndrome (MeSH terms) | Cost-benefit analysis (MeSH Terms) |
| Obesity, morbid (MeSH Terms) | Cost-effectiveness analysis (MeSH Terms) |
| Weight problems (tiab) | Economic evaluation (tiab) |
| Excess weight (tiab) |  |
| ‘’Over weight’’ (tiab) |  |
| Excess fat (tiab) |  |

((((((((((((obesity[MeSH Terms]) OR (overweight[MeSH Terms])) OR (body mass index[MeSH Terms])) OR (obesity, abdominal[MeSH Terms])) OR (abdominal obesity metabolic syndrome[MeSH Terms])) OR (obesity, morbid[MeSH Terms])) OR (obes*[Title/Abstract])) OR (BMI[Title/Abstract])) OR (weight problems[Title/Abstract])) OR (excess weight[Title/Abstract])) OR (''over weight''[Title/Abstract])) OR (excess fat[Title/Abstract])) AND (((((((((cost of illness[MeSH Terms]) OR (economics[MeSH Terms])) OR (costs and cost analysis[MeSH Terms])) OR (health care costs[MeSH Terms])) OR (health expenditure[MeSH Terms])) OR (cost-benefit analysis[MeSH Terms])) OR (cost-effectiveness analysis[MeSH Terms])) OR (economic outcomes[Title/Abstract])) OR (economic evaluation[Title/Abstract]))

Filters for search 02/05/2023: English, human, MEDLINE, 2016/1/1 – 2023/5/2

Hits: 3145

Filters for search 19/12/2024: English, human, MEDLINE, 2023/5/2 – 2024/12/19

Hits: 576

**Search term list Embase, Ovid** (5, 32, 36, 40)

Keyword table 02/05/2023 and 19/12/2024

| **Keyword 1** | **Keyword 2** |
| --- | --- |
| Obesity | Cost of illness |
| Obes* | Economics |
| Overweight | Economic outcomes |
| BMI | Costs and cost analysis |
| Body mass index | Health care costs |
| Obesity, abdominal | Health expenditures |
| Abdominal obesity metabolic syndrome | Cost-benefit analysis |
| Obesity, morbid | Cost-effectiveness analysis |
| Weight problems | Economic evaluation |
| Excess weight |  |
| Over weight |  |
| Excess fat |  |

1. (obesity OR obes* OR overweight OR BMI OR body mass index OR obesity, abdominal OR abdominal obesity metabolic syndrome OR obesity, morbid OR weight problems OR excess weight OR over weight OR excess fat).ab,ti 960003
2. *"obesity"/ 210472
3. "obesity"/ 529492
4. "body mass"/ 616822
5. "Obesity".kw. 110497
6. 1 OR 2 OR 3 OR 4 OR 5 1220221
7. (cost of illness OR economics OR economic outcomes OR costs and cost analysis OR health care costs OR health expenditures OR cost-benefit analysis OR cost-effectiveness analysis OR economic evaluation).ab,ti 68646
8. "Cost of illness".kw. 2013
9. *"cost of illness"/ 6262
10. *"health care cost"/ 48368
11. 7 OR 8 OR 9 OR 10 115884
12. 6 AND 11 3777
13. Limit 12 to English, human, journal, (article or article in press), and 2016-2023 1281

For updated search 13 is: Limit 12 to English, human, journal, (article or article in press), and 2023-2024 421

**Search term list PsycINFO** (32, 36, 40, 72)

Keyword table 02/05/2023 and 19/12/2024

| **Keyword 1** | **Keyword 2** |
| --- | --- |
| Obesity | Cost of illness |
| Obes* | Economics |
| Overweight | Economic outcomes |
| BMI | Costs and cost analysis |
| Body mass index | Health care costs |
| Obesity, abdominal | Health expenditures |
| Abdominal obesity metabolic syndrome | Cost-benefit analysis |
| Obesity, morbid | Cost-effectiveness analysis |
| Weight problems | Economic evaluation |
| Excess weight |  |
| Over weight |  |
| Excess fat |  |

1. TI ( (obesity OR obes* OR overweight OR BMI OR body mass index OR obesity, abdominal OR abdominal obesity metabolic syndrome OR obesity, morbid OR weight problems OR excess weight OR over weight OR excess fat) ) OR AB ( (obesity OR obes* OR overweight OR BMI OR body mass index OR obesity, abdominal OR abdominal obesity metabolic syndrome OR obesity, morbid OR weight problems OR excess weight OR over weight OR excess fat) ) 70107
2. KW obesity 22558
3. DE ‘’Body Mass Index’’ 18910
4. DE ‘’Obesity’’ 33773
5. 1 OR 2 OR 3 OR 4 76155
6. TI ( (cost of illness OR economics OR economic outcomes OR costs and cost analysis OR health care costs OR health expenditures OR cost-benefit analysis OR cost-effectiveness analysis OR economic evaluation) ) OR AB ( (cost of illness OR economics OR economic outcomes OR costs and cost analysis OR health care costs OR health expenditures OR cost-benefit analysis OR cost-effectiveness analysis OR economic evaluation) ) 135219
7. KW Cost of illness 237
8. DE ‘’Health Care Costs’’ 13072
9. 6 OR 7 OR 8 141295
10. 5 AND 9 2756
11. Limit 10 to English, peer reviewed journal (publication type), journal article (document type), and 2016-2023 695

For updated search 11 is: Limit 10 to English, peer reviewed journal (publication type), journal article (document type), and 2023-2024 113

**Search term list Econlit** (32, 36, 40, 72)

Keyword table 02/05/2023 and 19/12/2024

| **Keyword 1** | **Keyword 2** |
| --- | --- |
| Obesity | Cost of illness |
| Obes* | Economics |
| Overweight | Economic outcomes |
| BMI | Costs and cost analysis |
| Body mass index | Health care costs |
| Obesity, abdominal | Health expenditures |
| Abdominal obesity metabolic syndrome | Cost-benefit analysis |
| Obesity, morbid | Cost-effectiveness analysis |
| Weight problems | Economic evaluation |
| Excess weight |  |
| Over weight |  |
| Excess fat |  |

1. TI ( (obesity OR obes* OR overweight OR BMI OR body mass index OR obesity, abdominal OR abdominal obesity metabolic syndrome OR obesity, morbid OR weight problems OR excess weight OR over weight OR excess fat) ) OR AB ( (obesity OR obes* OR overweight OR BMI OR body mass index OR obesity, abdominal OR abdominal obesity metabolic syndrome OR obesity, morbid OR weight problems OR excess weight OR over weight OR excess fat) ) 3438
2. KW ‘’obesity’’ 1443
3. 1 OR 2 3443
4. TI ( (cost of illness OR economics OR economic outcomes OR costs and cost analysis OR health care costs OR health expenditures OR cost-benefit analysis OR cost-effectiveness analysis OR economic evaluation) ) OR AB ( (cost of illness OR economics OR economic outcomes OR costs and cost analysis OR health care costs OR health expenditures OR cost-benefit analysis OR cost-effectiveness analysis OR economic evaluation) ) 369753
5. 3 AND 4 875
6. Limit 5 to English, journal article (type), and 2016-2023 211

For updated search 6 is: Limit 5 to English, journal article (type) and 2023-2024 49

**Total hits from May search in 4 databases together 🡪 5305**

**Total hits from December search in 4 databases together 🡪 1159**
