# Supplementary material for: Costs of mental health care resource use in people with obesity: A systematic review
Source: PLoS One. 2025 Oct 8;20(10):e0333123. doi: 10.1371/journal.pone.0333123 (PMC12507204; doi:10.1371/journal.pone.0333123)
Supplement: S2 Table — (DOCX) [file pone.0333123.s003.docx]

# S3 Table. Data extraction sheet general characteristics

| **Author, Publication, Year, Country *** | **Title *** | **Research objective *** | **Population *** | **Comparator (if mentioned) *** | **Condition (how did they define obesity) *** | **Time horizon** | **Primary or secondary data** | **What is/are the data source(s) used to estimate the costs *** | **Synthesise of data regarding costs** |
| --- | --- | --- | --- | --- | --- | --- | --- | --- | --- |
| M. Aboulghate et al., 2021, Egypt | The Burden of Obesity in Egypt | Estimating the burden of obesity to society is an essential step in setting priorities and raising awareness. We aimed to assess the clinical, humanistic and economic burden of obesity for adults in Egypt. | Adult population in Egypt | N.M. | WHO classification | 1 year | Primary and secondary | Expert interviews and publications and payers' services list. | Top–down approach, retrospective, prevalence-based |
| E. Andersson et al., 2022, Sweden | Current and future costs of obesity in Sweden | To estimate the costs of obesity and to make a prognosis for the costs of obesity in 2030. | Swedish adults ≥ 25 years | Nonobese people | WHO classification | 1 year | Secondary | Statistics, public register data, and published scientific studies and reports. | Top–down approach, retrospective, prevalence-based |
| J. Ard et al., 2023, United States | Disease Burden and Health Status among People with Severe Obesity Who Do Not Receive Bariatric Surgery: A Retrospective Study | To compare eligible individuals who were or were not treated with bariatric surgery and describe disease burden, treatment, and healthcare costs over 3 years in individuals who did not undergo surgery. | Individuals were required to be at least 18 years old, and to be eligible for bariatric surgery according to current US guidelines (obesity class II [BMI 35.0–39.9 kg/m2] and ≥1 severe ORC or obesity class III [BMI ≥40.0 kg/m2]) | Individuals who underwent bariatric surgery versus those who did not. | Class II obesity (BMI 35.0–39.9 kg/m² with at least one obesity-related complication) or class III obesity (BMI ≥40.0 kg/m²). | 3 years | Secondary | Data from the IQVIA Ambulatory Electronic Medical Records – US database, linked to the US-based IQVIA PharMetrics® Plus administrative claims database. | Bottom-up approach, retrospective, prevalence-based |
| V. Atella et al., 2023, Italy | Outpatient healthcare costs associated with overweight and obesity in Italy | To evaluate outpatient healthcare expenditure associated with different levels of BMI and glucose metabolism alterations. | 991,917 adults in Italy | Within each BMI category, comparisons were made between normal glucose tolerance (NGT), impaired fasting glucose (IFG), and diabetes mellitus (DM) | BMI ≥ 30 kg/m², classified as class 1 (30-34.99), class 2 (35-39.99), and class 3 (≥40) | 1 year | Secondary | Health Search/IQVIA Health LPD Longitudinal Patient Database (HS), an Italian general practice registry, containing data from electronic clinical records (ECRs) of patients aged 14 + registered with a group of 900 General Practitioners (GPs), distributed across all Italian regions to be representative of the Italian population of GP’s patients. | Bottom-up, retrospective, prevalence-based |
| V. Atella et al., 2024, Italy | Lifetime costs of overweight and obesity in Italy | To estimate the lifetime cost profiles of different BMI classes (normal weight, overweight, and obesity I, II, III) in a primary care setting​ | Patients aged 14+ registered with a group of 800 GPs, distributed in all Italian regions to be representative of the Italian population of GPs. | Normal weight individuals | Normal weight: BMI 18.5–24.99 Overweight: BMI 25–29.99 Obesity class 1: BMI 30–34.99 Obesity class 2: BMI 35–39.99 Obesity class 3: BMI ≥40 | Lifetime, data from 14 years | Secondary | Health Search/IQVIA Health LPD Longitudinal Patient Database, an Italian general practice registry | Bottom-up, retrospective, incidence-based |
| N. Black et al., 2018, Australia | The Health Care Costs of Childhood Obesity in Australia: An Instrumental Variables Approach | To estimate the effect of childhood obesity on healthcare costs incurred by the Australian government using an instrumental variables approach​ | Children aged 6 to 13 | Normal weight children | BMI categories were based on international age- and gender-specific cut-points | 8 years | Secondary | Medicare administrative records, including the Medicare Benefits Schedule (MBS) and Pharmaceutical Benefits Scheme (PBS) | Bottom-up approach, retrospective, prevalence-based |
| M. Borges et al., 2024, Portugal | Burden of disease and cost of illness of overweight and obesity in Portugal | To estimate the disease burden and healthcare costs associated with overweight and obesity in the adult population of mainland Portugal for the year 2018. | Adult population aged 18–84 years in mainland Portugal. | N.M. | N.M. | 1 year | Secondary | National Health Survey with Physical Examination (NHSPE) 2015 and the National Food, Nutrition and Physical Activity Survey. | Top-down approach, retrospective, prevalence-based |
| S. Butalia et al., 2023, Canada | Health care cost of severe obesity and obesity-related comorbidities: A retrospective cohort study from Alberta, Canada | To estimate the incremental healthcare costs associated with severe obesity and its related health conditions in Alberta, Canada, during a one-year observation period. | Adults who underwent a procedure in Alberta, Canada, with a body mass index (BMI) ≥ 35 kg/m² (investigational cohort) and those with a BMI < 35 kg/m² (control cohort). | The control cohort, consisting of individuals with a BMI < 35 kg/m². | Obesity (body mass index [BMI] ≥ 30 kg/m2) and severe obesity (BMI ≥ 35 kg/m2) | 1 year | Secondary | Administrative health data from Alberta, Canada. | Top-down approach, retrospective, prevalence-based |
| A. Colao et al., 2017, Italy | Healthcare usage and economic impact of non-treated obesity in Italy: findings from a retrospective administrative and clinical database analysis | To investigate the prevalence of obesity in Italy and examine its resource consumption and economic impact on the Italian National Healthcare System (NHS) | All patients aged ≥18 years with at least one recorded body mass index (BMI) measurement between January 1, 2009, and December 31, 2012, from three health units in Northern (Bergamo, Lombardy), Central (Grosseto, Tuscany), and Southern (Naples, Campania) Italy. | Normal weight subjects (BMI < 25 kg/m²). | WHO classification | 1 year | Secondary | Primary care data, medical prescriptions, specialist consultations, and hospital discharge records from 2009 to 2013. | Top-down approach, retrospective, prevalence-based |
| K. Destri et al., 2024, Portugal | Hospitalization costs in Portugal among people with obesity: results from a nationwide population-based cohort 2011 to 2021 | To investigate the association between obesity and hospitalizations in the Portuguese adult population and compare the average costs of hospitalization among participants with and without obesity. | 10,102 participants aged ≥18 years from the Portuguese population | Normal weight individuals | WHO classification | 10 years | Secondary | Hospitalization records and associated costs are categorized according to national legislation and valued based on Diagnosis Related Groups pricing. | Top-down approach, prospective, prevalence-based |
| T. Effertz et al., 2016, Germany | The Costs and Consequences of Obesity in Germany: A New Approach from a Prevalence and Life-Cycle Perspective | To estimate the social costs of obesity in Germany. | A sample of 146,000 insured persons from the Techniker Krankenkasse (TK). Half of the sample constituted the "treatment" group with harmful consumption patterns, and the other half comprised individuals without such a diagnosis. | Nonobese people | WHO classification | 1 year | Secondary | Dataset from the German Statutory Health Insurance (SHI) | Bottom-up approach, retrospective, prevalence-and-incidence-based |
| Y. Gil-Rojas et al., 2019, Colombia | Burden of Disease Attributable to Obesity and Overweight in Colombia | To estimate the burden of disease attributable to obesity and overweight conditions using disability-adjusted life-years (DALYs) in Colombia. | The entire Colombian population in 5-year periods: younger than 5, 5 to 14, 15 to 49, 50 to 60, and older than 70 years. | N.M. | WHO classification | 1 year | Secondary | A literature review. Price information of the medicines was obtained from the System of Prices of Medicines report and the drug price regulation newsletters published by the Ministry of Health. The costs of procedures, laboratories, and consultations were obtained from the Social Security Institute 2001 tariff. | Top–down approach, retrospective, prevalence-based |
| V. Gorasso et al., 2022, Belgium | Health care costs and lost productivity costs related to excess weight in Belgium | To estimate the annual health care and lost productivity costs associated with excess weight among the adult population in Belgium. | Adult individuals in Belgium | Normal weight individuals | WHO classification | 1 year | Secondary | Belgian Health Interview Survey (BHIS) 2013 and individual health insurance data from 2013 to 2017. | Top-down approach, retrospective, prevalence-based |
| J. Hecker et al., 2022, The Netherlands | Burden of disease study of overweight and obesity; the societal impact in terms of cost-of-illness and health-related quality of life | To examine the societal burden of overweight and obesity on the Dutch population in terms of cost-of-illness (COI) and HRQoL. | 97 individuals ≥18 years with overweight or obesity. | N.M. | WHO classification | 6 months, but extrapolated results to 1 year | Primary and secondary | Primary data: A questionnaire: The TIC-P , that gives insights into general information and costs. Secondary data: Dutch guidelines and Statistics Nederland | Bottom-up, prospective, prevalence-based |
| P. Kamble et al., 2018, United States | Association of obesity with healthcare resource utilization and costs in a commercial population | To examine the association of obesity with healthcare resource utilization (HRU) and costs among commercially insured individuals. | Commercially insured adults in the United States. | Normal weight individuals | WHO classification | 4 years | Secondary | Commercial health insurance claims database. | Top-down approach, retrospective, prevalence-based |
| S. Kent et al., 2017, Engeland | Hospital costs in relation to body-mass index in 1·1 million women in England: a prospective cohort study | To examine the association between BMI and hospital costs among women in England. | 1,093,866 women aged 50–64 years at recruitment, participating in the NHS breast cancer screening programme | Women with a BMI of 20.0 kg/m² to less than 22.5 kg/m². | WHO classification | Average following of 4.9 years | Secondary | Hospital Episode Statistics (HES) data, which includes information on hospital admissions and procedures. | Top–down approach, prospective, prevalence-based |
| J. Kjellberg et al., 2017, Denmark | The socioeconomic burden of obesity | To evaluate the socioeconomic impact of obesity by estimating the direct and indirect costs associated with obesity in Denmark. | A representative sample of a pooled population older than 16 years from The Danish National Health Profile 2010 and 2013 with BMI > 30 kg/m2. | N.M. | WHO classification | 1 year | Secondary | The Danish National Health Profile 2010 and 2013, International Statistical Classification of Diseases and Related Health Problems 10th Revision, the National Patient Registry, National Health Insurance Service Register. | Top–down approach, retrospective, prevalence-based |
| L. Kompaniyets et al., 2020, United States | Hospital Length of Stay, Charges, and Costs Associated With a Diagnosis of Obesity in US Children and Youth, 2006-2016 | To analyse the hospital LOS, charges, and costs associated with an obesity diagnosis among children aged 2–19 years. | ∼4.1 million discharges of children aged 2–19 years during 2006–2016, from the National Inpatient Sample (NIS) database. | Nonobese 2-19 year olds | They do not define obesity directly in the text, but they do reference an article from Centers for Disease Control and Prevention about childhood obesity facts and there the definition is: Obesity is defined as a body mass index (BMI) at or above the 95th percentile of the CDC sex-specific. | 10 years | Secondary | Population from National Inpatient Sample (NIS) database. ICD9 and ICD10 used for diagnoses. To identify primary conditions that co-occur with the secondary diagnosis of obesity, we use the Clinical Classifications Software (CCS) tool that clusters the ICD diagnoses into distinct categories. | Top–down approach, retrospective, prevalence-based |
| K.E. Kyler et al., 2023, United States | Medicaid Expenditures among Children with Documented Obesity | 1. Describe the total annual health care expenditures for a sample of Medicaid enrolled children with documented obesity 2. Identify characteristics associated with high spending within the cohort 3. How inpatient and outpatient mental health spending contributes to overall spending for children with documented obesity | 300,286 children aged 2–17 years, from the 2017 MarketScan Medicaid database | N.M. | Included all ICD-10-CM codes for obesity. This wasn't elaborated to define obesity specifically | 1 year | Secondary | Cost data available in the MarketScan Medicaid database for fee-for-service insurance plans. Capitated insurance plan data was not available therefore, standardised payments for all claims were calculated using methods described by Kuo, et al (2015) and Bettenhausen, et al. (2018) | Top-down, retrospective, prevalence-based |
| S.T. Lartey et al., 2020, Ghana | Health service utilization and direct healthcare costs associated with obesity in older adult population in Ghana | Examine the associations among health service utilization, healthcare costs and excess weight in the older adult population of Ghana in 2014/15 | Sub-sample of 3350 respondents aged 50 years or older with complete responses from the World Health Organization’s Study on global AGEing and adult health (WHO-SAGE) Wave 2 | Underweight, normal weight older patients | WHO classification | 1 year | Primary and secondary | WHO SAGE questionnaire | Bottom-up, retrospective, prevalence-based |
| S. Musich et al., 2016, United States | The Impact of Obesity on Health Care Utilization and Expenditures in a Medicare Supplement Population | Estimate magnitude of BMI (from self-reported height and weight) on healthcare utilisation and expenditure in a group of older adults with Medicare Supplement plans | Random sample of 9,484 respondents aged >=65 years old from 10 US states with enrollment in AARP Medicare Supplement plan | N.M. | Underweight (BMI at or below 18.5), normal weight (BMI = 18.6-24.9), overweight (BMI = 25-29.9), and obese (BMI = 30 or greater). Definition from National Heart, Lung, and Blood Institute (NHLBI) and Obesity Task Force (1998) | Assumed 12 months follow-up | Secondary | Medicare claims | Bottom-up, retrospective, prevalence-based |
| J. Pearson-Stuttard et al., 2024, United Kingdom | Variations in healthcare costs by body mass index and obesity-related complications in a UK population: A retrospective open cohort study | Identify and characterize the subgroup of individuals with obesity who incurred the highest costs in 2019 | "Eligible individuals aged 18 years or older with known sex" | N.M. | Overweight (25–<30 kg/m2), obesity class I (30–<35 kg/m2 ), obesity class II (35–<40 kg/m2 ), obesity class III (≥40 kg/m2) according to NICE guidance | Mean/median follow-up not reported. Maximum 15 years (total study period) | Secondary | Primary care: Personal Social Services Research Unit (PSSRU, 2020) Secondary care: Secondary Uses Service by National Health Service Digital Prescriptions: calculated using net ingrdient costs fromnational report | Bottom-up, retrospective, prevalence-based |
| X, Qin et al., 2016, China | The Medical Cost Attributable to Obesity and Overweight in China: Estimation Based on Longitudinal Surveys | Estimate medical cost attributable to obesity and overweight in China | 14,615 individuals representing 77 urban and 146 rural communities | N.M. | WHO classification | Cross-sectional cost estimates over a period of 9 years | Secondary | 2000-2009 CHNS (China Health and Nutrition Survey) | Bottom-up approach, retrospective, prevalence-based |
| S. Reitzinger et al., 2024, Austria | Low-, moderate-, and high-risk obesity in association with cost drivers, costs over the lifecycle, and life expectancy | Estimate the societal burden of the three obesity classes (low-, moderate- and high-risk) in the Austrian population and identify the respective cost drivers and the effects on life expectancy | Representative sample from Austrian Health Interview survey for adults; Austrian Child Obesity Surveillance Initiative for girls aged 5-19 and boys aged 5-14; military service medical examination data for boys aged 15-19; German data from under 5s (total n. observations not reported) | N.M. | Adults: Class 1 obesity (BMI 30–34.9), class 2 obesity (BMI 35–39.9), class 3 obesity (BMI ≥ 40). Children: Obese or not obese | Lifetime | Secondary | COI database. Indirect costs based on Austrian gross income data, employment-rate data and disability pension scheme | Top-down, retrospective, incidence-based |
| C. Rudisill et al., 2016, England | Are healthcare costs from obesity associated with body mass index, comorbidity or depression? Cohort study using electronic health records | Investigate the association between body mass index (BMI) category and healthcare costs, focusing on the issue of whether BMI category, obesity-related comorbidity and/or depression most strongly determines costs related to obesity | 250,046 adults aged >=20 from a random sample of CPRD only English GPs (~50k per BMI category), excluding patients who have had bariatric surgery | N.M. | BMI categories: 18.5–24.9; 25.0–29.9; 30.0–34.9; 35.0–39.9; >=40.0 | 5 year | Secondary | PSSRU (2013) for healthcare costs; RESIP UK dictionary for prescription costs | Bottom-up, retrospective, prevalence-based |
| H.J. Song et al., 2018, South Korea | The impact of obesity and overweight on medical expenditures and disease incidence in Korea from 2002 to 2013 | Evaluate the impact of BMI on medical expenditures and disease incidence and prevalence over more than 10 years in South Korea using a national longitudinal database from 2002–2013 | 496469 participants from the NHIS-HEALS database (excludes cancer patients, pregnant women and individuals with BMI>60) | Entire population': individuals with BMI data at baseline (2002-03) 'Consistent BMI level population': BMI remained in same category at baseline and observation end date (2012-23) | According to Asian criteria, BMI was defined as underweight (<18.5 kg/m2), normal weight (18.5–22.99), overweight (23–24.99), obesity I (25–29.99), obesity II (30–34.99), and obesity III (35–59.99) | 11 years | Secondary | NHIS-HEALS database | Bottom-up, retrospective, prevalence-based |
| M. Spanggaard et al., 2022, Denmark | The substantial costs to society associated with obesity-a Danish register-based study based on 2002-2018 data | Conduct a large-scale study estimating the burden of obesity, the associated costs of various comorbidities, and the breakdown of associated direct and indirect costs in Denmark using BMI registrations from the Danish national health registers. | Adults registered on the Danish National Patient Register (NPR) between 2002-18 with a primary or secondary diagnosis of obesity in a hospital | 5 non-obese unique controls for each person with obesity were randomly selected from the general population via the Danish Civil Registration System (CRS), matched on age, gender, education, and region of residence in the index year | WHO classification | Average follow-up not reported, data collected retrospectively from 2002-18 | Secondary | Healthcare: NPR database Medications: Danish National Prescription Registry | Bottom-up, retrospective, prevalence-based |
| D. Steinl et al., 2024, Switzerland | Cost of overweight, obesity, and related complications in Switzerland 2021 | Provide a current estimate of the direct costs associated with managing overweight and obesity, including treatment of related complications, among adolescents (≥15 years) and adults in Switzerland | Adolescents (15+) and adults with overweight/obesity - extrapolated from 2017 SHS to >3.1 million people | N.M. | Overweight/obese defined as BMI >=25 | 1 year | Secondary | Costs calculated for each comorbidity, all taken from different sources | Varied |
| B.T. Suehs et al., 2017, United States | Association of obesity with healthcare utilization and costs in a Medicare population | To examine the association of obesity with healthcare resource utilisation and costs in a Medicare population | 172,866 individuals aged >=65 years old covered by Medicare Advantage Prescription Drug (MAPD) plan with at least one medical claim between 2008-2012 | N.M. | Normal (BMI 19 to 24.9, V85.1), overweight (BMI 25 to 29.9, V85.2x), obese class I (BMI 30 to 34.9, V85.30–V85.34), obese class II (BMI 35 to 39.9, V85.35–V85.39), and obese class III categories (BMI >=40, V85.4) | Minimum 24 months | Secondary | MAPD Humana Inc. database | Bottom-up, retrospective, prevalence-based |
| A. Vesikansa et al. 2023, Finland | Obesity and Metabolic State Are Associated with Increased Healthcare Resource and Medication Use and Costs: A Finnish Population-Based Study | To characterise healthcare resource (HCRU) and medication use and associated costs in individuals with obesity compared with individuals with normal weight or overweight in a population-based cohort of | 5587 FinHealth 2017 study participants who had BMI measured in the health examination available and who were not underweight (BMI<18.5 kg/m2). The study cohort has been described in more detail  elsewhere. | Healthy weight and overweight | WHO classification | 1 year | Secondary | National FinHealth 2017 health examination survey (participants), Data on healthcare visits and hospital stays, including diagnoses (ICD-10), and purchases and costs of prescription medicines were collected from the nationwide registers by the Finnish Institute for Health and Welfare and Social Insurance Institution of Finland. The healthcare costs were calculated based on standard unit costs reported by the Finnish Institute for Health and Welfare | Top–down approach, retrospective, prevalence-based |
| A.H. Wijga et al., 2018, The Netherlands | Healthcare utilisation and expenditure of overweight and non-overweight children | Explore the potential of linking birth cohort data with administrative data to assess the utilisation and costs of healthcare of overweight and non-overweight children | 1453 participants aged 11-14 from the PIAMA birth cohort | Non-overweight vs overweight (child must be same category at age 11 and age 14) | Not explicitly stated; based on International Obesity Task Force (IOTF) age- and sex-specific criteria | 1 year | Secondary | Vektis national database | Bottom-up, retrospective, prevalence-based |
| K. Williamson et al., 2023, Scotland | Overlooked and under-evidenced: Community health and long-term care service needs, utilization, and costs incurred by people with severe obesity | Document individual-level community health and LTC service usage for an exemplar cohort of people with severe obesity | 25 participants aged 40-87, BMI >=40 and receiving care services | N.M. | Severe obesity BMI >=40kg/m2 | 1 year (plus any one-off longer term costs for equipment and adaptations) | Primary | PSSRU and King's Fund (information in Supplm) | Bottom-up, retrospective, prevalence-based |
| N. Yates et al., 2016, Germany | The Economic Burden of Obesity in Germany: Results from the Population-Based KORA Studies | To estimate the excess costs of obese compared to normal-weight persons in Germany based on self-reported resource utilisation and work absence. | 9,070 observations, containing 6,731 individuals aged 31-96 years. All participants of KORA are of German nationality and have been selected from Augsburg, a city in the south-west of Germany, and two surrounding districts. | Healthy weight | WHO classification | 1 year | Secondary | Data from five cohort surveys of the KORA project (Cooperative Health Research in the Augsburg Region) were pooled. These surveys were performed in 2004 to 2005 (F3), 2006 to 2008 (F4), 2008 to 2009 (Age 1), 2010 (FoLu) and 2012 (Age 2). | Bottom-up approach, retrospective, prevalence-based |

# * = This data were summarised verbatim based on what the authors mentioned in their article. N.M. = Not mentioned
